# Non-ossifying Fibroma Pathological Fracture in a Patient With Lactose Intolerance

**DOI:** 10.7759/cureus.17225

**Published:** 2021-08-16

**Authors:** Khaled Alshehri, Alshahid A Fadil

**Affiliations:** 1 Orthopedic Surgery, King Fahad Medical City, Riyadh, SAU

**Keywords:** non-ossifying fibroma, pathological fracture, non-ossifying fibroma surgery recovery, lactose intolerance, pediatric pathological fracture

## Abstract

Non-ossifying fibroma (NOF) is a frequently occurring benign tumor of children and adolescents. In the long bones, it appears as an eccentric, expanded lesion in the metaphyseal diaphyseal area. Most cases are asymptomatic and resolve at a later age while others might become symptomatic and have a high risk of fracture. We present a case of a 15-year-old boy who is known to have lactose intolerance, suffered a pathological fracture following trauma, and was diagnosed with non-ossifying fibroma of the proximal tibia. The etiology of these lesions is not well-known. However, there might be a relation between tendons and NOF. This reported case of NOF is in the proximal tibia, which is a common site of the lesion beside the distal femur. Our reported case was treated by open curettage and grafting, which is the recommended classical treatment. On follow-up, full union was achieved without complications.

## Introduction

Non-ossifying fibroma (NOF) is considered a benign lesion of long bones and is usually asymptomatic. Etiology is still controversial on whether it should be considered a benign lesion or a developmental disorder of long bones in adolescents. Most patients require no treatment. But pathological fractures have been reported in the literature [1]. There have been descriptions of non-ossifying fibroma associated with neurofibromatosis, Jaffe-Campanacci syndrome, and hypogonadism [2-3]. No reported cases have been published on the relation between lactose intolerance and NOF. We present a case of a 15-year-old lactose-intolerant boy with pathological tibial fracture of the non-ossifying fibroma. A bone graft was done in the form of cancellous chips and putty with internal fixation of the fracture. This treatment showed satisfactory results with progressive healing of the upper tibial transverse fracture without complication.

## Case presentation

**Figure 1:**
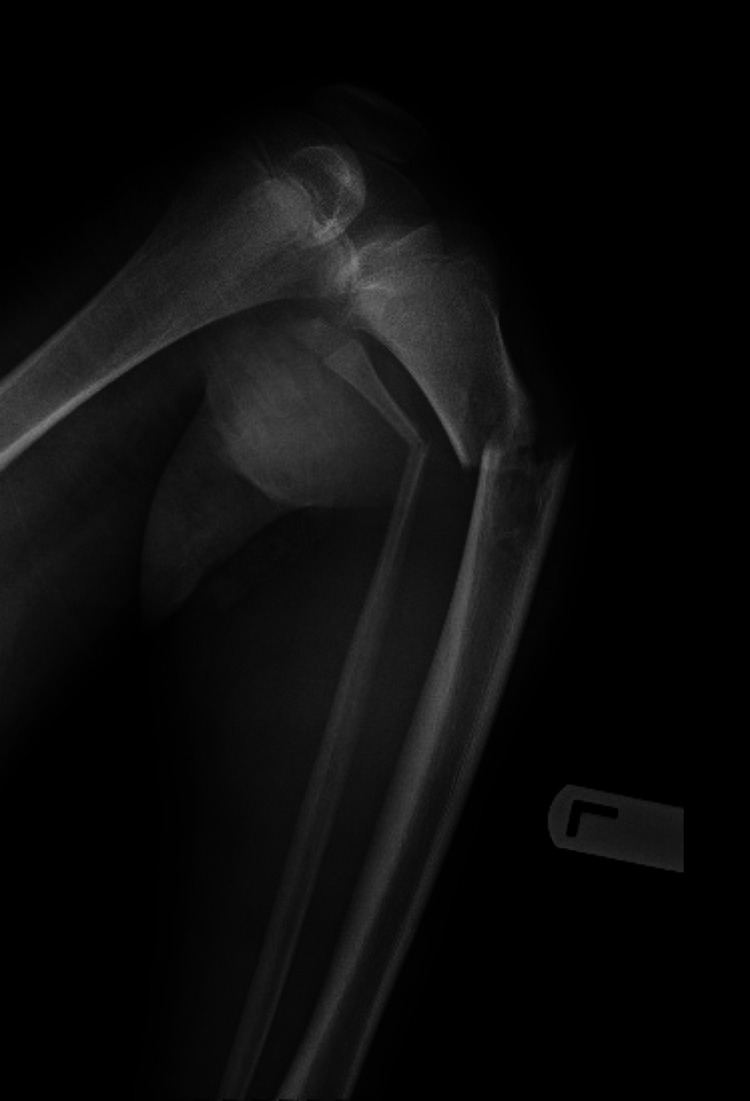
X-ray of the left leg on initial presentation

This is a 15-year-old boy known to have congenital lactase deficiency. He presented to our emergency department with an isolated injury to the left leg, having pain, swelling, and deformity after falling in a football game. There was no previous significant history. Clinically, the fracture was closed and the distal neurovascular examination was intact with soft compartments. The patient was active, playing football, and attending regular school on a special diet given his medical condition. Plain film radiographs of the left knee showed an angulated pathological fracture through the proximal tibia and fibula metaphysis (Figure 1).

**Figure 2:**
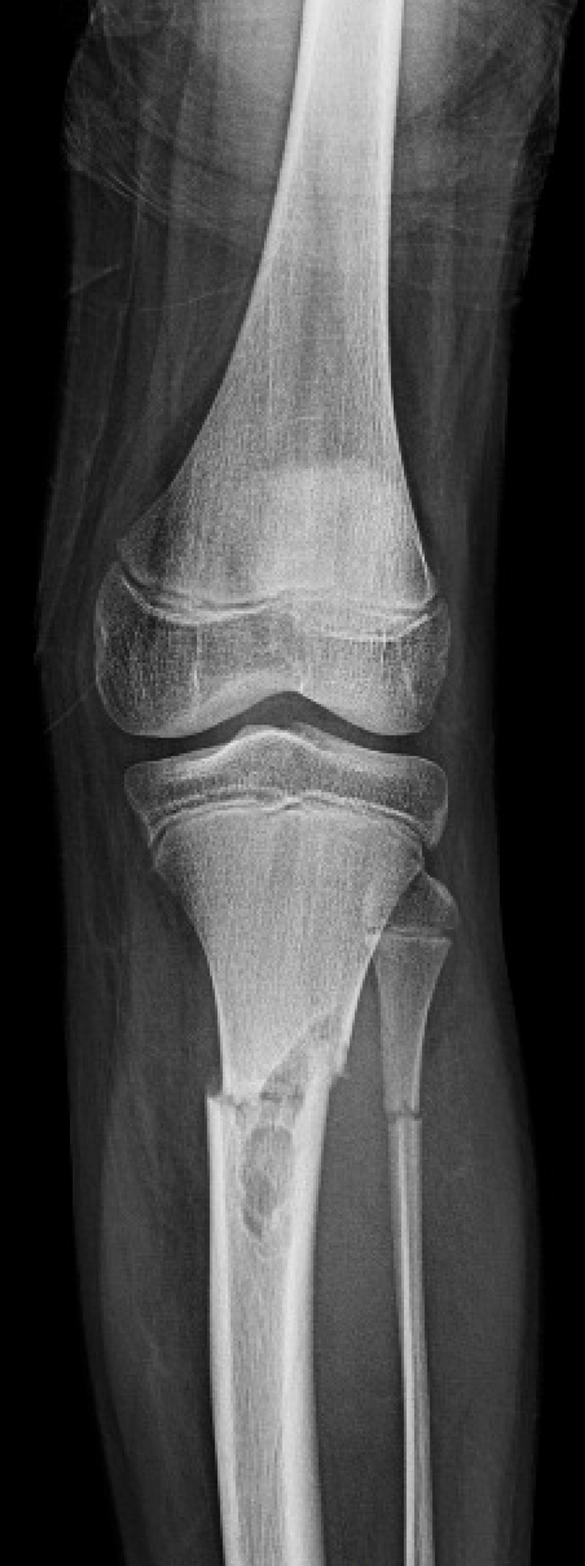
AP view of the left tibia and fibula fracture AP: anteroposterior

**Figure 3:**
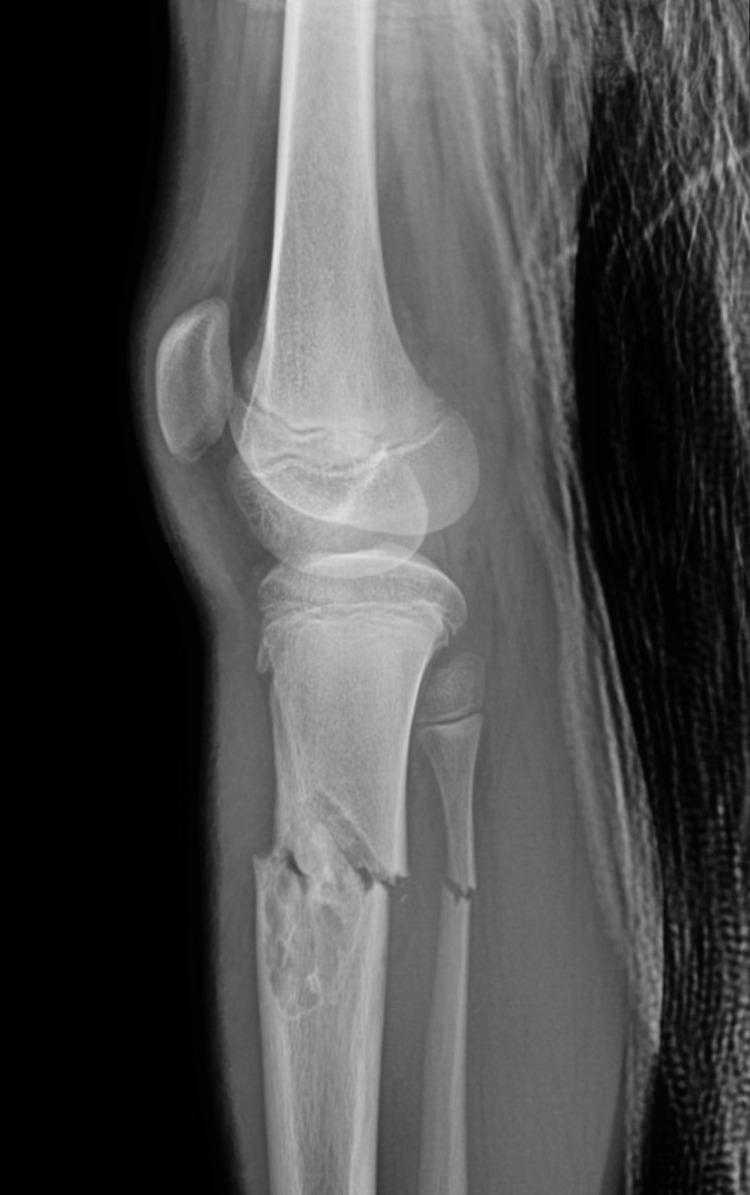
Lateral view of the left tibia fibula fracture

The fracture was passing through a multilobulated lucent eccentric lesion with faint sclerotic margins involving the tibia (Figures 2-3).

Surgery was performed after signed consent. The patient was informed that data concerning his case would be submitted for publication. His consent was obtained and signed by his father.

Treatment

Upon consultation in the emergency room (ER), closed reduction was performed under conscious sedation in acceptable alignment in preparation for surgery back slab applied (Figures 2-3). The distal neurovascular exam was normal all through. The skeletal survey was negative for any other lesion. Based on history, clinical, and radiological findings, the diagnosis of NOF was reached and confirmed with the senior musculoskeletal (MSK) radiologist. Blood investigations were unremarkable.

**Figure 4:**
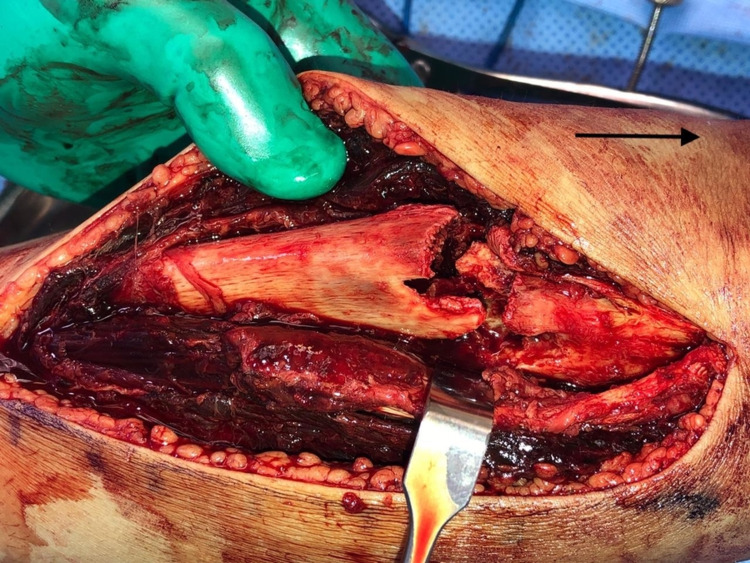
Intraoperative image showing the pathological fracture (arrow pointing toward the head)

**Figure 5:**
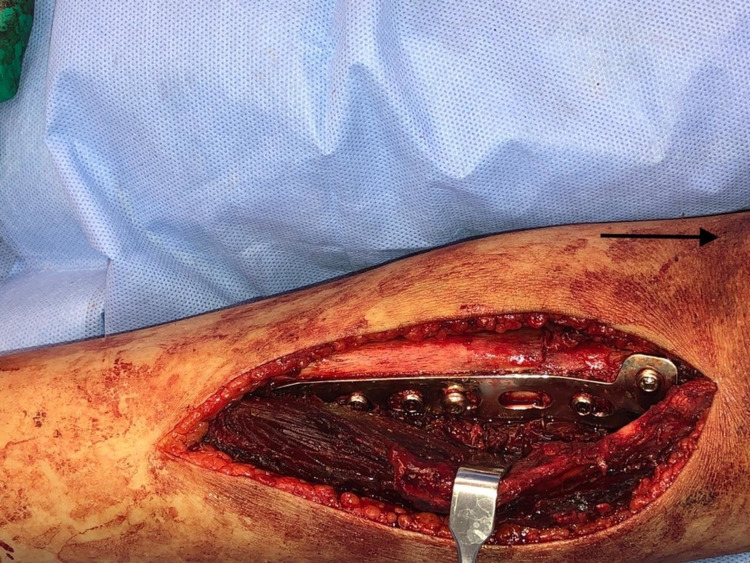
Intraoperative image showing the pathological fracture after fixation and bone grafting (arrow pointing toward the head)

**Figure 6:**
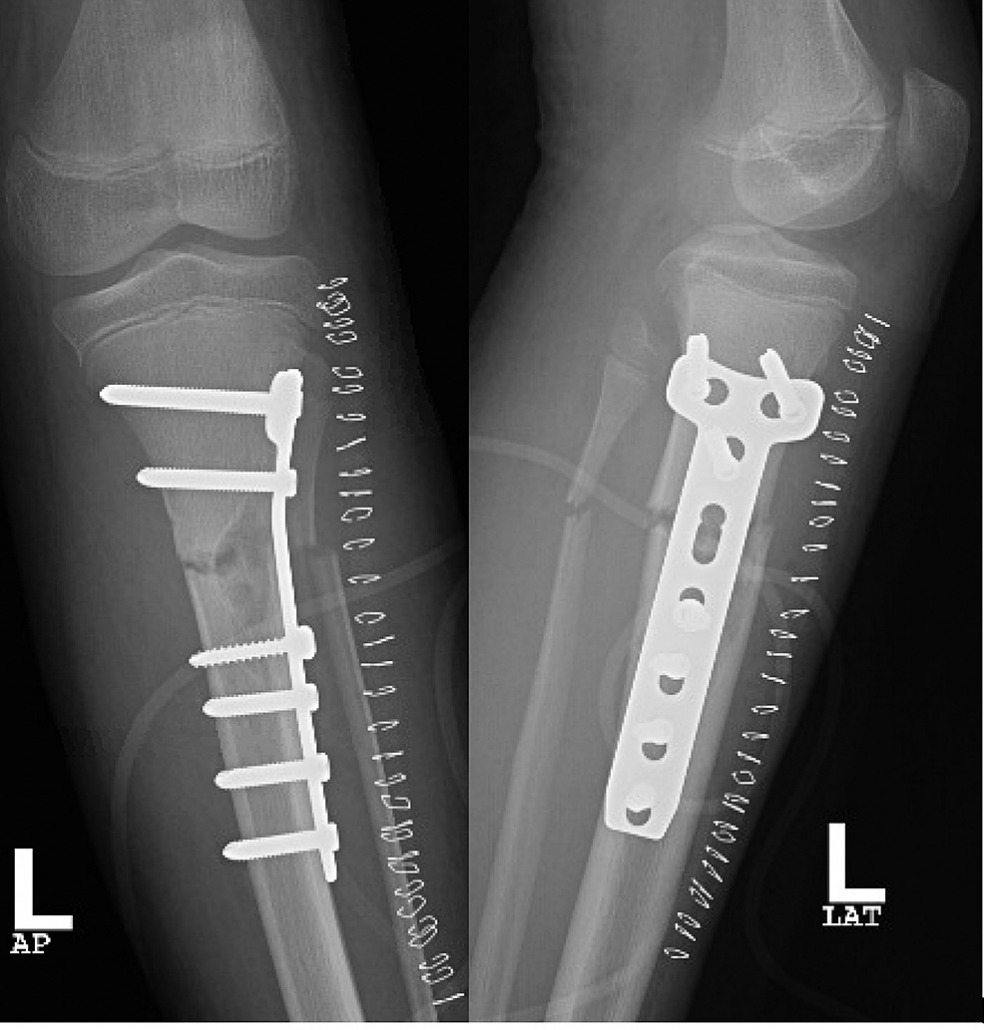
AP and lateral views of the left tibia post fixation and bone grafting AP: anteroposterior

The standard anterolateral approach was utilized, exposing the fracture site and obtaining an open biopsy (Figure 4), followed by thorough irrigation and debridement. Using a 3.5-mm locking plate, reduction and fixation were achieved. Bone graft was used in the form of cancellous chips and putty (Figures 5-6), The patient was discharged on a back slab to restrict weight-bearing.

Follow-up

Three Weeks Follow-Up

The wound was assessed and the clips removed. Histopathology was reported as non-ossifying fibroma.

Two Months Follow-Up

**Figure 7:**
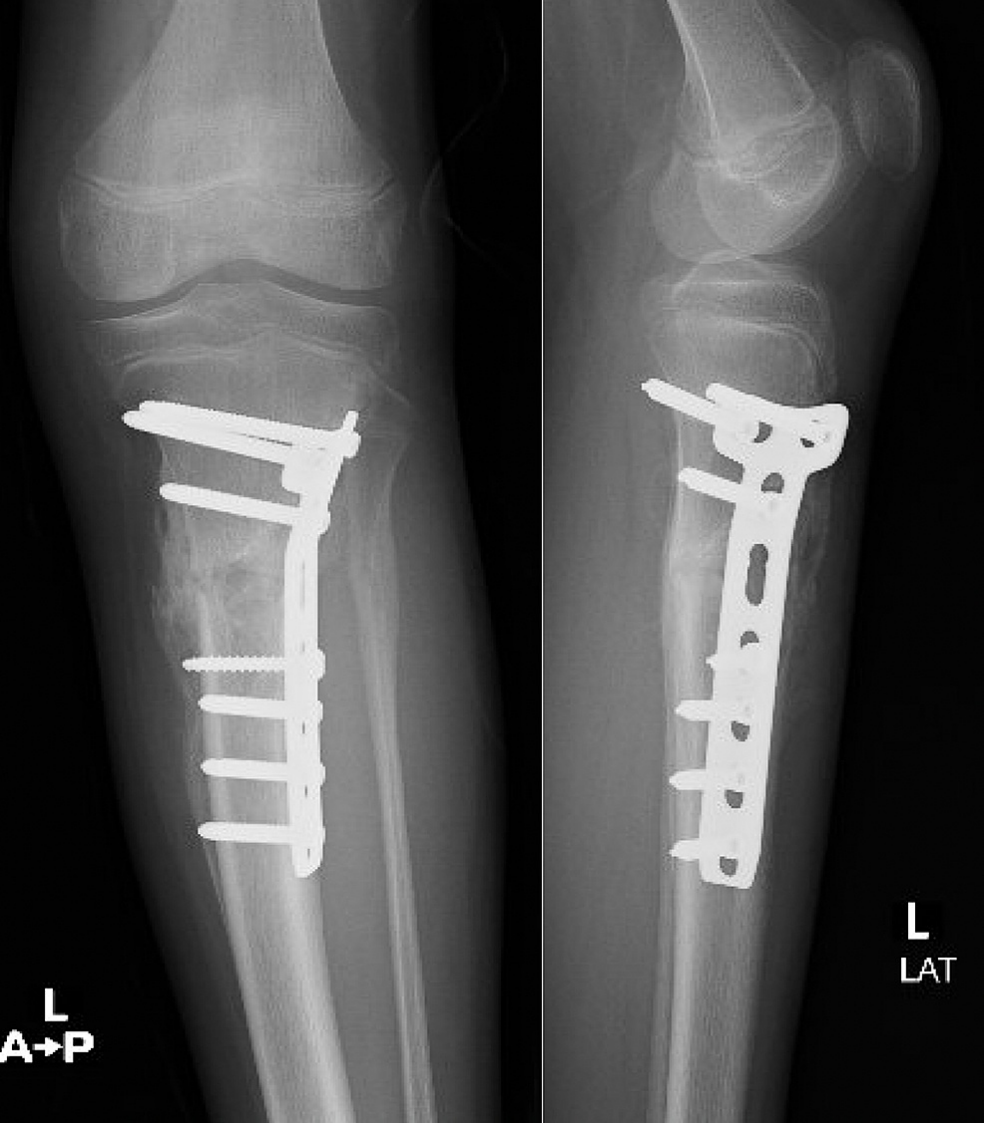
AP and lateral views of the left tibia showing healing at the fracture site AP: anteroposterior

He was on partial weight-bearing. There was no pain and progressive healing of the upper tibial fracture (Figure 7).

## Discussion

Non-ossifying fibroma (NOF) is a common benign tumor of the fibrous tissue, which usually occurs in the first or second decade of life. In most cases, the lesion resolves at the age of 20-25 years. The diagnosis of NOF is based on radiographs [4]. Stress type fractures are also present in NOF in skeletally immature [5].

The etiology of NOF is not clearly defined. Some theories state that they form from a disturbance of the physis or from the bone marrow cell lineage. Some studies propose the localization of the lesion in children is based on the traction of the interosseous membrane. The external rotation of the fibula with respect to the tibia might generate traction [6].

Ritschl et al. classified NOF into four stages. Stage A characterizes the small lesion near the growth plate. In Stage B, the grape-shaped, thin sclerotic borders of the lesion are present, and it lies distant from the growth plate. In Stage C, the sclerosis increases, and the mineralization of the bone starts from the shaft toward the growth plate. Stage D is characterized by complete homogeneous sclerosis of the lesion. Stage B is related to a high risk of bone fractures [7].

Arata et al. reviewed 23 pathological fractures caused by NOF. Among all fractures, only one was present at a location other than the lower extremity. The average age when the fracture occurs was found to be 12 years. About 50% of the bone was occupied by the lesion in the anterior-posterior and lateral planes. The vertical length was about 33 mm in all cases. Hence, lesions correlating to the above sizes should be monitored closely, as they have an increased risk of fracture [8].

Most of the NOF are asymptomatic while some are large and can cause fracture of the long bones and chronic pain [9-10]. The NOF diagnosis is done based on radiographs and clinical presentation. The disease usually presents with an asymptomatic multilobulated lesion in a random radiograph.

In our study, the boy suffered from the displaced pathological fracture through the proximal tibia and fibula metaphysis of the left lower limb by falling. Most NOF-associated fractures occur in the metaphysis in the long bones [11].

The lesion has fibroblast proliferation along with the multinucleated giant cells that resemble osteoclasts [12]. Most cases of NOF occur in the long bones such as the metaphysis of the femur and tibia. Non-ossifying fibroma X-ray has a characteristic pattern, and it resolves on its own, especially in the cases of smaller lesions. However, if there is a pathologic fracture or the risk of fracture is present, there should be a consideration for surgical intervention [13].

The phenomenon of progression of the lesion to fracture is unclear. However, Goldin et al. presented a system of classification that might predict the fracture risk in a particular lesion. The classification is based on the point allotted according to the findings on the computed tomography (CT) scan: (1) the coronal view shows greater than 50% of the width; (2) the sagittal view shows greater than 50% of the view; (3) the breach in the cortex; (4) no neocortex is present. The study concluded that the higher the point on the CT scan, the higher will be the risk of fracture.

MRI is rarely needed in diagnosis because nearly every lesion is clearly seen in CT scans. However, radiation exposure in CT scan is inappropriate for young patients. In radiographs, the lesion appears as lucent, hazy, and indistinct margins while the cortex may appear as thinned or expanded [14]. Along the long axis of the bone, the greatest length of the lesion can be determined. The lesions appearing in radiographs might be in different phases; some might be inactive while others might be in the involution phase [8].

Sakamoto et al. analyzed 44 cases of NOF. Among 47 lesions, they found 45 lesions of the lower extremity while only two lesions were present in the upper limbs. About 21% of the cases had a lesion greater than 4 cm while 32% had lesion expansion at the cortex. Larger-sized lesions were observed and 17% of the cases presented lesions on the proximal tibia. About 50% of the cases were present in the distal tibia. Hence, most cases of larger lesions are present on the distal and proximal metaphysis of the tibia [15].

In our case, we used bone grafting and curettage as a classical treatment approach. However, these classical surgeries failed to cure the critical cases of NOF. Parwaz et al. studied pathological fractures of the femur, which were nonunion even after the two surgeries. In this critical case, vascularized fibular graft provided mechanical cortical support along with regenerative potential. Hence, vascularized fibular graft might be a new possible treatment for a more challenging union [16].

The relationship of the tendons with NOF is also considered by Goldin et al. They found that the NOFs of the distal femur originate from medial and lateral gastrocnemius. The lesions might have an origin from the physis or metaphysis. However, the lesions don’t attach to the physis. The NOF lesions in long bones migrate along the diaphysis as the patient grows. The fibroblast production in the tendon helps increase the matrix metalloproteinases production similar to the bone [17].

Our patient presented with NOF along with lactose intolerance. Lactose-containing dairy food is the most common source of lactose. In adults, the lactase enzyme deficiency leads to intolerance to this disaccharide [18]. The focused therapy for lactose intolerance is to restrict the food containing lactose. Lactose-reduced products, prebiotics, plant-based dairy substitutes, and exogenous lactose are different methods of treatment. However, in the English literature, there is no case reported linking NOF to lactose intolerance [19].

## Conclusions

Non-ossifying fibroma is a benign neoplasm of fibrous tissue in children or adolescents. It usually affects the long bones around the knee. The classical treatment plans are bone graft and curettage for those who have large lesions and pathological fractures. In our case, bone-grafting showed progressive healing of the upper tibial transverse fracture. Our literature review also found no evidence between lactose intolerance and NOF. Further studies are required for the advanced diagnostic and treatment options of the lesion.
